# Editorial: Cardiovascular involvement in autoimmune diseases

**DOI:** 10.3389/fcvm.2022.982559

**Published:** 2022-07-25

**Authors:** Sophie I. Mavrogeni, Lambros Fotis, Paraskevi V. Voulgari, Aliki I. Venetsanopoulou, Marco Mattucci-Cerinic

**Affiliations:** ^1^Onassis Cardiac Surgery Center, Athens, Greece; ^2^Attikon Hospital, Athens, Greece; ^3^Department of Internal Medicine, Rheumatology Clinic, Medical School, University of Ioannina, Ioannina, Greece; ^4^Department of Experimental and Clinical Medicine, Division of Rheumatology, Azienda Ospedaliero Universitaria Careggi, University of Florence, Florence, Italy

**Keywords:** autoimmune rheumatic diseases, cardiovascular disease, biomarkers, early cardiac involvement, myocardial fibrosis, cardiac magnetic resonance

Autoimmune rheumatic diseases (ARDs) is a group of diseases in which tolerance to self-antigens and/or immunoregulation is compromised and leads to inappropriate immune reactivity against body tissues, including the heart. The most prevalent ARDs presenting with significant cardiovascular disease (CVD) are: rheumatoid arthritis (RA) and spondyloarthropathies, systemic lupus erythematosus, systemic vasculitides, inflammatory myopathies, mixed connective tissue diseases, systemic sclerosis, Sjogren syndrome as well as sarcoidosis, a multisystem granulomatous disorder. Although new targeted treatments against ARDs led in significant improvement of disease-associated mortality, ARD patients still have a lower average life expectancy, compared with the general population (1), mainly due to the increased incidence of (CVD) (2).

Irrespective of etiology, CVD in patients with ARDs may remain asymptomatic or with only few, atypical symptoms. As a result, clinically overt CVD is discovered at a late stage, and carries an ominous prognosis (3). European league against rheumatism (EULAR) proposed principles emphasizing the need of regular screening, management of modifiable CVR factors and the endorsement of patient education (4). However, in these guidelines the crucial role of cardiovascular imaging in the assessment of CVD in ARDs is not discussed. It seems that rheumatologists are mainly based on clinical evaluation and blood biomarkers to decide about the involvement of the CV system. However, CVD imaging is the “sine qua non” approach to reveal early CV alterations and response to treatment, representing a “direct assessment” of CV status, beyond blood biomarkers.

Echocardiography, nuclear techniques and computed tomography have been already applied for CVD evaluation of ARDs. Recently, cardiac magnetic resonance (CMR) proved its superiority in CVD evaluation of patients with ARDs, due to the concurrent assessment of function, angiography and tissue characterization in the same examination. This gives the opportunity to the clinicians to have a direct “glimpse” of pathophysiologic phenomena taking place in CV system, before overt clinical presentation will appear, even in cases, where blood biomarkers are still normal (5). CMR by allowing the performance of oedema/fibrosis imaging can detect the differentiation between recent/active and chronic CV lesions and guide further risk stratification and treatment individualization (5).

Until now, the CV diagnosis in patients with ARDs had a long exhaustive journey through “Scylla and Charybdis” of clinical evaluation, which quite often is unable to differentiate between true CV and other musculoskeletal symptoms. The clear knowledge of the clinical evaluation, electrocardiogram and blood biomarkers limitations, in conjunction with the tremendous development of CV imaging opens new horizons in the diagnosis, risk stratification and treatment evaluation of patients with ARDs. CV imaging can provide an objective, reproducible way to assess the CV status and therefore, it should be incorporated in the Rheumatology guidelines, as an important parameter for decision making. Similarly to ancient Odysseus, who found his way to Ithaca after many adventures, CVD in patients with ARDs must find its own way to early diagnosis and treatment individualization. However, this way needs great clinical awareness and excellent knowledge of all available diagnostic tools.

In this issue of Frontiers in Cardiovascular Medicine, we publish papers referring to CVD approach in patients with ARDs. The use of circulating biomarkers for the screening of cardiac alterations in RA patients, the use of CMR to evaluate early cardiac involvement and myocardial fibrosis in ARD are presented among others in this issue articles, showing that the currently available noninvasive tools have the power to lead “CVD in patients with ARDs” to Ithaca of early, accurate diagnosis/treatment individualization and contribute to the reduction of morbidity/mortality, due to CVD.

## Author contributions

All authors contributed to the critical review and revision of the manuscript and approved the final version.

## Conflict of interest

The authors declare that the research was conducted in the absence of any commercial or financial relationships that could be construed as a potential conflict of interest.

## Publisher's note

All claims expressed in this article are solely those of the authors and do not necessarily represent those of their affiliated organizations, or those of the publisher, the editors and the reviewers. Any product that may be evaluated in this article, or claim that may be made by its manufacturer, is not guaranteed or endorsed by the publisher.
